# The Relationship Between Blood Omega‐3 Levels and the Small Vessel Disease in Ischemic Stroke Patients: A Case‐Control Study

**DOI:** 10.1002/hsr2.70652

**Published:** 2025-04-21

**Authors:** Leila Afshar Hezarkhani, Kamran Mansouri, Nader Salari, Mojtaba Ammari‐Allahyari, Hasti Shahbazi, Milad MohamadYari, Masoud Mohammadi

**Affiliations:** ^1^ Neuroscience Research Center, Health Technology Institute Kermanshah University of Medical Sciences Kermanshah Iran; ^2^ Medical Biology Research Center, Health Technology Institute Kermanshah University of Medical Sciences Kermanshah Iran; ^3^ Department of Molecular Medicine, School of Medicine Kermanshah University of Medical Sciences Kermanshah Iran; ^4^ Sleep Disorders Research Center Kermanshah University of Medical Sciences Kermanshah Iran; ^5^ Department of Biostatistics, School of Health Kermanshah University of Medical Sciences Kermanshah Iran; ^6^ Business Department, Academic Development University of Sunderland London UK; ^7^ Department of Neurology, School of Medicine Kermanshah University of Medical Sciences Kermanshah Iran; ^8^ Department of Neurology, Imam Reza Hospital Kermanshah University of Medical Sciences Kermanshah Iran; ^9^ Research Center for Non‐Communicable Diseases Jahrom University of Medical Sciences Jahrom Iran

**Keywords:** ischemic stroke, small vessel disease, stroke

## Abstract

**Background:**

Stroke is one of the leading causes of death worldwide, and it can lead to the development of small vessel disease in the brain, which in turn exacerbates the unintended and long‐term consequences of this condition. Given that various factors are involved in the development and exacerbation of this disease, the aim of the present study is to determine the relationship between blood omega‐3 levels and the small vessel disease in ischemic stroke patients.

**Methods:**

The present study is a case‐control study. The target population in this study consists of ischemic stroke patients enrolled in a hospital through a census over the course of 1 year. The sample size was 72 patients, who were assessed using MRI and/or CT scans for the extent of small vessel blood vessel changes. They were then divided into two groups: those with small vessel changes (31 individuals—cases) and those without small vessel changes (41 individuals—controls). Subsequently, individuals in both groups were evaluated for their blood omega‐3 levels. Statistical analysis was conducted using SPSS‐20.

**Results:**

The research findings revealed that the majority of individuals in both the case and control groups were in the 51–70 age range and were female. Apart from the gender variable, the two groups did not significantly differ in other variables. Upon comparing the two groups, it was evident that men comprised a larger portion of the case group. Analytical findings also indicated a significant relationship between blood omega‐3 levels and small vessel changes in the brain. This is because the results of the mean blood omega‐3 levels of the two case and control groups showed a statistically significant difference between the two groups (*p* = 0.001 < 0.05).

**Conclusion:**

Based on the research findings, there is an association between low levels of omega‐3 in blood and the occurrence of small vessel changes in the brain.

## 1 Background

Ischemic stroke, characterized as infarction in the brain, spinal cord, or retina, accounts for 71% of all strokes globally [1]. Both ischemic and hemorrhagic strokes annually affect approximately 13.7 million people worldwide, making it the second leading cause of death, causing 5.5 million deaths annually [2]. It is estimated that one in every four adults experiences a stroke in their lifetime, with more than 80 million stroke survivors worldwide [3]. Various pathological processes affecting the structure or function of small arteries, capillaries, or venules in the brain, and causing substantial damage to cerebral arteries and venules, as well as impacting subcortical white and gray matter, are collectively referred to as small vessel disease of the brain [4].

Non‐modifiable risk factors for small vessel disease of the brain and the occurrence of ischemic stroke include age, gender, and genetic factors. A more rapid increase in the risk of ischemic stroke after the age of 49 has been observed, with a higher prevalence after the age of 39 in developed countries compared to developing countries [5]. According to the Global Burden of Disease study, ischemic stroke is more common in men than in women [6]. Several modifiable risk factors for ischemic stroke have been identified, including high blood pressure (with a threshold of 90/160 mmHg), low levels of regular physical activity, a high apolipoprotein B to A ratio, dietary factors, waist‐to‐hip ratio, psychosocial stress and depression, smoking, cardiac causes (such as atrial fibrillation and prior myocardial infarction), excessive alcohol consumption, and diabetes mellitus [7].

A reverse relationship between fish consumption and the risk of stroke has been reported in numerous studies [8, 9], although not all past studies have shown such an association [10, 11]. Fish consumption creates a protective mechanism against stroke by preventing platelet aggregation [12, 13], lowering blood pressure [14], reducing insulin resistance [15], and reducing plasma fibrinogen [16]. It is possible that fish consumption may increase the risk of hemorrhagic strokes. Ecological studies were conducted on the indigenous population in Greenland, which consumed high levels of omega‐3 polyunsaturated fatty acids (omega‐3 PUFAs) through fish intake. These studies found a higher mortality rate from hemorrhagic strokes in comparison to the Danish population [17]. Studies have demonstrated that fish oil rich in N‐3 PUFAs can reduce the cardiovascular and cerebrovascular diseases [18]. Moreover, the omega‐3 fatty acids in the diet may reduce insulin resistance and glucose intolerance [15], potentially lowering the risk of lacunar infarctions since glucose intolerance and diabetes are strongly associated with this event [19, 20].

Considering the severe and often chronic consequences of stroke, including small vessel disease of the brain, and the heavy burden it imposes on the healthcare system, individuals, families, and communities, it is necessary to investigate all factors that can contribute to the reduction of this disease [2, 3]. Among these factors, the potential role of omega‐3 in reducing strokes is of great significance [18]. Due to the lack of sufficient studies in this area and its significant importance, we decided to conduct a study with the aim of determining the relationship between blood omega‐3 levels and the small vessel disease in ischemic stroke patients.

## 2 Methods

This study is a matched case‐control study with a purpose to investigate the relationship between the small vessel disease and blood omega‐3 levels in ischemic stroke patients. The ethical approval for this study was obtained from Kermanshah University of Medical Sciences (IR.KUMS. REC.1399.936).

The study population included all patients who were hospitalized due to ischemic stroke in the Neurology Department of one hospital in Kermanshah during the year 2021–2022. The inclusion criteria for the study was a diagnosis of ischemic stroke and informed patient consent. We enrolled patients who were diagnosed with a first‐episode, non‐cardioembolic ischemic stroke mechanism and admitted to our hospital within 7 days after symptom onset. All of the patients were evaluated by CT scan. Patients with evidence of severe small vessel diseases (Fazekas score equal or more than 2) were enrolled in the case group. Other patients were evaluated by MRI, and the participants with evidence of SVD in MRI were added to the case group, and patients with no evidence of SVD in MRI were enrolled in the control group. The sampling method in this study was continuous census over the course of 1 year, and a total of 72 patients were included in the study. After evaluating the patients using MRI and/or CT scans to assess the extent of small vessel blood vessel changes, they were divided into two groups: those with small vessel changes (31 individuals) and those without small vessel changes (41 individuals). Subsequently, data collection began.

## 3 Measure

Following informed consent from participants, demographic characteristics (patient age, gender, occupation, education, marital status), medical and clinical history (body mass index, age of disease onset, type of medication used, average daily medication dosage, duration of medication use, type of accompanying medication, average daily dosage of accompanying medication, duration of accompanying medication use, blood pressure, diabetes, lipids, cholesterol, smoking, smoking history, substance use, alcohol consumption, family history of physical illness, type of physical illness in the family), and laboratory results (triglycerides, lipids, cholesterol, blood pressure, BMI, FBS, LDL, HDL, BUN, Cr) of patients were examined and recorded from their medical records and through patient interviews. The diagnosis and assessment of omega‐3 among patients involved a comprehensive process. This process evaluated blood plasma phospholipids and expressed them as a percentage of total phospholipid fatty acids based on weight for measuring the phospholipid. Firstly, plasma total lipids were extracted using the Folch method. Then, the phospholipid fraction was isolated through a thorough thin‐layer chromatography process using a development solvent composed of hexane, diethyl ether, and acetic acid (80:20:2). Subsequently, the phospholipid fractions were methylated to FA methyl esters (FAMEs) using the Lepage and Roy method. The FAMEs of individual FAs of phospholipids were then separated by gas chromatography using a model 6890 apparatus (Agilent Technologies, Palo Alto, CA, USA) with a 30 m Omegawaz TM 250 capillary column (Supelco, Bellefonte, PA, USA). Peak retention times were obtained by comparison with known standards (37 component FAME mix and PUFAs‐2, Supelco; GLC37, NuCheck Prep, Elysian, MN, USA) and analyzed with ChemStation software (Agilent Technologies). Finally, plasma phospholipid FAs were expressed as the percentage of total FAs, and the mean value was calculated from duplicate measurements of each sample. The diagnosis and assessment of omega‐3 among patients were determined by a comprehensive process, evaluating blood plasma phospholipids and expressing them as a percentage of total phospholipid fatty acids based on weight for measuring the phospholipid. Plasma total lipids were extracted according to the Folch method, and the phospholipid fraction was isolated by a thin‐layer chromatography process using a development solvent composed of hexane, diethyl ether, and acetic acid (80:20:2). The phospholipid fractions were then methylated to FA methyl esters (FAMEs) by the Lepage and Roy method. The FAMEs of individual FAs of phospholipids were separated by gas chromatography using a model 6890 apparatus (Agilent Technologies, Palo Alto, CA, USA) with a 30 m Omegawaz TM 250 capillary column (Supelco, Bellefonte, PA, USA). Peak retention times were obtained by comparison with known standards (37 component FAME mix and PUFAs‐2, Supelco; GLC37, NuCheck Prep, Elysian, MN, USA) and analyzed with ChemStation software (Agilent Technologies). Plasma phospholipid FAs were expressed as the percentage of total FAs. The mean value was calculated from duplicate measurements of each sample.

## 4 Data Analysis

After collecting and entering the data into the software, data analysis was performed using SPSS 20. Qualitative variables were described in terms of frequency and percentage, while quantitative variables were described in terms of mean, standard deviation, and range of variation.

To compare variables between the case and control groups, the *χ* ^2^ test was used for qualitative variables, and independent *t*‐tests or one‐way analysis of variance (ANOVA) were used for quantitative variables. Logistic regression was used to control confounding factors.

## 5 Results

The current study was conducted on 72 ischemic stroke patients, with 31 ischemic patients without small vessel changes in the control group (Group 1) and 41 ischemic patients with small vessel changes in the case group (Group 2). Demographic characteristics (age, gender, occupation, education, marital status) and self‐reported histories (smoking, substance use, alcohol consumption) are reported in Table 1. Except for the gender variable, the two groups did not show significant differences in other variables. It was observed that men comprised a larger portion of the case group compared to the control group.

**Table 1:** Comparison of the frequency distribution of demographic characteristics among study groups.

Factor	Levels	Control group *N* = 31	Case group *N* = 41	Total	*χ* ^2^ (significance level)
Current age	50 >	11 (35.5)	9 (22.0)	20 (28.7)	5.690 (0.26)
	51–70	13 (41.9)	16 (39.0)	29 (40.3)	
	> 70	7 (22.6)	16 (39.0)	23 (31.9)	
Sex	Male	10 (32.3)	23 (56.1)	33 (45.8)	4.041* (0.03)
	Female	21 67.7)	18 (43.9)	39 (54.2)	
Education level	Illiterate/elementary school	9 (29.0)	13 (31.7)	22 (30.6)	6.982 (0.72)
	High school	11 (35.5)	18 (43.9)	29 (40.3)	
	Post‐secondary education and above	11 (35.5)	10 (24.4)	21 (29.2)	
Occupation	Unemployed	1 (3.2)	5 (12.2)	6 (8.3)	6.110 (0.14)
	Self employed	9 (29.0)	20 (48.8)	29 (40.3)	
	Government	4 (12.9)	5 (12.2)	9 (12.5)	
	Housekeeper	17 (54.8)	11 (26.8)	28 (38.9)	
Marital status	Single	3 (9.7)	3 (7.3)	6 (8.3)	0.351 (0.83)
	Married	26 (83.9)	34 (82.9)	60 (83.3)	
	Widowed	2 (6.5)	4 (9.8)	6 (8.3)	
Smoking	Yes	18 (58.1)	26 (63.4)	44 (61.1)	0.213 (0.64)
	No	13 (41.9)	15 (36.6)	28 (38.9)	
Drug use	Yes	6 (19.4)	13 (31.7)	19 (26.4)	1.378 (0.18)
	No	25 (80.6)	28 (68.3)	53 (73.6)	
Alcohol consumption	Yes	4 (12.9)	8 (19.5)	12 (16.7)	0.55 (0.45)
	No	27 (87.1)	33 (80.5)	60 (83.3)	

* At a significance level of 0.05.

Table 2 reports the examination of medical and clinical histories of the patients. Based on this table, the two groups did not have a significant difference in terms of medical and clinical histories.

**Table 2:** Medical and clinical histories of patients.

Factor	Levels	Control group *N* = 31	Case group *N* = 41	Total	*χ* ^2^ (significance level)
The disease diagnosis age	< 50	15 (48.4)	12 (29.3)	27 (37.5)	2.924 (0.232)
	51–70	12 (38.7)	20 (48.8)	32 (44.4)	
	70 <	4 (12.9)	9 (22.0)	13 (18.1)	
High blood pressure disease	Yes	27 (87.1)	39 (95.1)	66 (91.7)	1.488 (0.214)
	No	4 (12.9)	2 (4.9)	6 (8.3)	
Hyperlipidemia	Yes	21 (67.7)	34 (82.9)	55 (76.4)	2.257 (0.111)
	No	10 (32.3)	7 (17.1)	17 (23.6)	
Diabetes	Yes	11 (35.5)	22 (53.7)	33 (45.8)	2.349 (0.098)
	No	20 (64.5)	19 (46.3)	39 (54.2)	
Hypercholesterolemia	No	(54.8) 171	24 (58.5)	41 (56.9)	0.638 (0.291)
	Yes	14 (45.2)	17 (41.5)	31 (43.1)	
Brain stroke history	No	27 (87.1)	32 (78.0)	59 (81.9)	0.977 (0.251)
	Yes	4 (12.9)	9 (22.0)	13 (18.9)	
Heart attack history	No	27 (87.1)	36 (87.7)	62 (87.3)	0.008 (0.921)
	Yes	4 (12.9)	5 (12.2)	9 (12.7)	
Triglycerides (mg/dL)	150 >	20 (64.5)	19 (46.3)	39 (54.2)	3.723 (0.155)
	151–199	7 (22.6)	9 (0.22)	16 (22.2)	
	200 <	4 (12.9)	13 (31.7)	17 (23.6)	
LDL (mg/dL)	130 >	13 (41.9)	14 (34.1)	27 (37.5)	2.555 (0.279)
	130–159	16 (51.6)	19 (46.3)	35 (48.6)	
	159 <	2 (6.5)	8 (19.5)	10 (13.9)	
HDL (mg/dL)	40 >	8 (25.8)	10 (24.4)	18 (25.0)	0.700 (0.705)
	41–60	20 (64.5)	29 (70.7)	49 (68.1)	
	60 <	3 (9.7)	2 (4.9)	5 (6.9)	
BUN (mg/dL)	6 >	6 (19.4)	6 (14.6)	12 (16.7)	0.676 (0.713)
	6–20	19 (61.3)	24 (58.5)	43 (59.7)	
	20 <	6 (19.4)	11 (26.8)	17 (23.6)	
Cr (mg/dL)	0.5 >	1 (2.3)	2 (4.9)	3 (4.2)	0.397 (0.820)
	0.5–1.3	28 (90.3)	35 (85.4)	63 (87.5)	
	1.3 <	2 (6.5)	4 (9.8)	6 (8.3)	
FBS (mg/dL)	> 70	1 (2.3)	0 (0.0)	1 (1.4)	2.148 (0.144)
	70–1110	20 (64.5)	23 (56.1)	43 (59.7)	
	110 <	10 (32.3)	18 (43.9)	28 (38.9)	
Blood pressure (mmHg)	80 >	0 (0.0)	0 (0.0)	0 (0.0)	1.925 (0.588)
	120–80	8 (25.8)	8 (19.5)	16 (22.2)	
	121–129	2 (6.5)	2 (4.9)	4 (5.6)	
	13–139	13 (41.9)	41 (34.1)	27 (37.5)	
	140 <	8 (25.8)	71 (41.5)	25 (34.5)	

The following table shows that in the case group, where all patients had some level of small vessel changes, 14 patients (34.1%) were in the “punctate” category, 15 patients (36.6%) were in the “beginning confluent” category, and 12 patients (29.3%) were in the “large confluent area” category. In the control group, all patients were devoid of any small vessel changes, and they all fell into the “absent” category regarding small vessel changes.

In the case group, for the 14 patients in the “punctate” category, the average blood omega‐3 level was reported as a mean of 4.59 (0.78%), and for the 15 patients in the “beginning confluent” category, the average blood omega‐3 level was reported as a mean of 4.22 (1.18%). For the 12 patients in the “large confluent area” category, the average blood omega‐3 level was reported as a mean of 3.73 (0.67%).

These results indicate that the average blood omega‐3 level of ischemic patients in the “punctate” small vessel changes category was higher than that of ischemic patients in the “beginning confluent” and “large confluent area” small vessel changes categories. However, the comparison of the average omega‐3 levels among these three categories did not show any statistically significant difference (*p* = 0.072 > 0.05), meaning that the average blood omega‐3 level of ischemic patients with different levels of small vessel changes did not have a statistically significant relationship.

In the control group, the average blood omega‐3 level of ischemic patients without small vessel changes was reported as a mean of 5.88 (1.52%). The results of these analyses are shown in the following table (Table 3).

**Table 3:** Comparison of blood omega‐3 levels in patients at different levels of small vascular changes in each of the case and control groups.

Groups	Statistics (significance)	Mean (SD)	Min–max	Number (percent)	Small vessel variation
Case group	Punctate (Fazekas 1)	14 (34.1)	3.75–6.10	4.59 (0.78)	2.83 (0.072)
	Beginning confluent (Fazekas 2)	15 (36.6)	3.10–8.00	4.22 (1.18)	
	Large confluent area (Fazekas 3)	12 (29.3)	2.65–4.50	3.73 (67.0)	
Control group	Absent	31 (100.0)	3.69–8.83	5.88 (1.52)	—

The average blood omega‐3 level in the case group with 41 ischemic patients with small vascular changes was reported as 4.21 (0.96%), while in the control group with 31 patients without small vascular changes, it was reported as 5.88 (1.52%). These results indicate that the average blood omega‐3 level in the case group is 1.68 units lower than the control group. The results of the comparison of the mean blood omega‐3 levels in patients between the two case and control groups showed a statistically significant difference (*p* = 0.0001 < 0.05), with the case group having significantly lower levels than the control group. These findings are presented in the following table (Table 4).

**Table 4:** Investigation and comparison of the mean blood omega‐3 levels in patients in the two case and control groups.

Groups	Statistics (significance)	Mean difference	Mean (standard deviation)	Median	Min–max	Number
Case group	41	2.65–6.00	4	4.21 (0.96)	1.68	5.73*
Control group	31	3.69–8.83	5	5.88 (1.52)		(0.0001)
Total patients	72	2.65–8.63	4.35	4.93 (1.48)	—	—

* At a significance level of 0.05.

In the regression model, the Wald statistical values and their significance levels indicated that only the presence of the omega‐3 factor in the logistic model is significant (*B* = −1.07, *T*_wald_ = 14.51, *p* < 0.0001). On the other hand, the results showed that in the logistic regression model, considering the value of the Wald statistical variable and its significance level, the gender factor (*B* = −1.07, *T*_Wald_ = 14.51, *p* = 0.164) did not have significant presence in the model (Table 5).

**Table 5:** Results of predicting the effect of omega‐3 blood levels and patient gender on the likelihood of developing small vessel changes in the studied ischemic patients using logistic regression.

Factors		*B*	SE	Wald (sig)	OR	Lower	Upper
—	Constant	5.951	1.42	17.66 (0.0001)*	384.07	—	—
Omega‐3 level	Omega‐3	−1.07	0.281	14.51 (0.0001)*	0.343	0.198	0.595
Sex	Male	−0.83	0.596	1.94 (0.164)	0.436	0.136	1.403

* At a significance level of 0.05.

## 6 Discussion

The current study was conducted with the aim of determining the relationship between blood omega‐3 levels and the small vessel disease in ischemic stroke patients. The results of the current study showed that the average blood omega‐3 level in the case group is 1.68 units lower than the control group. The comparison of the mean blood omega‐3 levels among the two case and control groups showed a statistically significant difference between the two groups (*p* = 0.0001 < 0.05), with the case group having a significantly lower mean than the control group.

In line with the current study, Song et al. also found in their study that low plasma omega‐3 levels are associated with pathological changes in small brain vessels in patients suffering from ischemic stroke. In the Song et al. study, conducted on an Asian population, low omega‐3 levels were linked not only to ischemic stroke but also to hemorrhagic stroke in SVD. Furthermore, low omega‐3 levels were associated with higher SVD scores, indicating its severity. Additionally, their study revealed that a diet rich in omega‐3 can reduce the disease burden and the severity of cerebral SVD, irrespective of hemorrhagic or ischemic characteristics [21]. Chowdhury et al., in their study, investigated the relationship between fish consumption, omega‐3 fatty acids, and the risk of cerebral vascular disease. They reported that individuals with high blood omega‐3 levels have a lower risk of cerebral vascular disease [22]. Another systematic study by Abdelhamid et al. in 2018 reported that the increase in omega‐3 levels, when having a beneficial effect on the outcomes, is only partially effective. They found strong evidence indicating that omega‐3 fatty acids reduce the risk of death from stroke in individuals with high omega‐3 consumption by 8.8% compared to the control group [23].

Based on the results of the study, individuals with ischemic stroke who have lower levels of omega‐3 are more likely to experience intracranial artery stenosis or occlusion compared to the healthy control group [24]. It can be argued that N‐3 polyunsaturated fatty acids (PUFAs), including eicosapentaenoic acid and docosahexaenoic acid, have potent anti‐inflammatory effects, reduce platelet aggregation, and prevent atherosclerotic plaque regression [25]. Ueno et al. also pointed out the effects of omega‐3 fatty acids on cerebral vascular changes in their systematic study in 2019 [25]. In another study by Bhat et al., the effect of omega‐3 in reducing cardiovascular diseases and strokes was confirmed [26]. Cupino et al. in a prospective cohort study also declared a reduced risk of developing cerebrovascular disease in individuals with lower consumption of N‐3 polyunsaturated fatty acids (PUFAs) than the control group [27]. The results of these studies are consistent with the current study [27].

Iso et al. found that increased fish consumption and omega‐3 intake are associated with a decreased ischemic stroke [28]. The results of the present study are supported by a study conducted by Park et al., which identifies low omega‐3 as a risk factor for ischemic stroke in Asian populations [29].

In the current study, most patients who experienced ischemic stroke with small vessel changes in the brain were male. This finding is consistent with the Ryu et al. study, which examined a population of 477 individuals with ischemic stroke. They observed that 61% of the participants were male [30]. In a study conducted by Ge et al., among 965 patients with ischemic stroke, 78.9% were male [31]. The Rawlley et al. study estimated the gender distribution in an interventional study and found that women comprised the majority of stroke patients. They attributed this result to health barriers for women and less frequent medical visits [32]. The results of this study do not align with the current study, which could be attributed to differences in sample size, study population, and study design.

One of the limitations of the current study was the data collection, which relied on self‐reporting by participants and information extraction from patients' medical records. However, the researchers aimed to minimize this bias by adhering to data confidentiality principles and collecting information from complete and unaltered medical records. Additionally, because this study was a case‐control study, there was a potential for recall bias in the participant interviews.

Despite these limitations, the current study has several strengths. Firstly, the study was conducted continuously over the course of 1 year, and all eligible patients were enrolled. All demographic, medical, and clinical variables were examined in both groups, with significant differences. The only variable that differed between the two groups, apart from omega‐3, was gender, which was investigated through logistic regression analysis.

## 7 Conclusion

Based on the present study's findings, a low level of omega‐3 in the blood is associated with SVD occurrence. Blood omega‐3 levels can predict the likelihood of developing SVD in patients with ischemic stroke. Omega‐3 can be introduced as a risk‐reducing or preventive factor for SVD. Therefore, as a first step, it is recommended to conduct further studies with larger sample sizes to strengthen the results. Given the importance of the role of omega‐3 in reducing this disease, it is advisable for individuals in the community to use dietary sources or pharmaceutical supplements containing omega‐3 as needed.

## Author Contributions

**Leila Afshar Hezarkhani:** conceptualization and investigation. **Kamran Mansouri:** conceptualization and investigation. **Nader Salari:** methodology, software, and formal analysis. **Mojtaba Ammari‐Allahyari:** supervision, writing – review and editing. **Hasti Shahbazi:** supervision, writing – review and editing, writing – original draft. **Milad MohamadYari:** writing – review and editing, writing – original draft. **Masoud Mohammadi:** methodology, writing – original draft, writing – review and editing, investigation.

## Ethics Statement

The authors have nothing to report.

## Conflicts of Interest

The authors declare no conflicts of interest.

## Transparency Statement

The lead author Masoud Mohammadi affirms that this manuscript is an honest, accurate, and transparent account of the study being reported; that no important aspects of the study have been omitted; and that any discrepancies from the study as planned (and, if relevant, registered) have been explained.

## Data Availability

The data that support the findings of this study are available from the corresponding author upon reasonable request.

## References

[1] V. L. Feigin , G. Nguyen , K. Cercy , et al., “Global, Regional, and Country‐Specific Lifetime Risks of Stroke,” New England Journal of Medicine 379, no. 25 (2018): 2429–2437. 30575491 10.1056/NEJMoa1804492 PMC6247346

[2] C. O. Johnson , M. Nguyen , G. A. Roth , et al., “Global, Regional, and National Burden of Stroke, 1990–2016: A Systematic Analysis for the Global Burden of Disease Study 2016,” Lancet Neurology 18, no. 5 (2019): 439–458. 30871944 10.1016/S1474-4422(19)30034-1 PMC6494974

[3] “WSO Global Stroke Fact Sheet,” World Stroke Organization, published 2022, https://www.world-stroke.org/news-and-blog/news/wso-global-stroke-fact-sheet-2022.

[4] J. C. Choi , “Genetics of Cerebral Small Vessel Disease,” Journal of Stroke 17, no. 1 (2015): 7. 25692103 10.5853/jos.2015.17.1.7 PMC4325630

[5] V. L. Feigin , R. V. Krishnamurthi , P. Parmar , et al., “Update on the Global Burden of Ischemic and Hemorrhagic Stroke in 1990–2013: The GBD 2013 Study,” Neuroepidemiology 45, no. 3 (2015): 161–176. 26505981 10.1159/000441085 PMC4633282

[6] V. L. Feigin , B. Norrving , and G. A. Mensah , “Global Burden of Stroke,” Circulation Research 120, no. 3 (2017): 439–448. 28154096 10.1161/CIRCRESAHA.116.308413

[7] M. J. O'Donnell , D. Xavier , L. Liu , et al., “Risk Factors for Ischaemic and Intracerebral Haemorrhagic Stroke in 22 Countries (The Interstroke Study): A Case‐Control Study,” Lancet 376, no. 9735 (2010): 112–123. 20561675 10.1016/S0140-6736(10)60834-3

[8] R. F. Gillum , M. Mussolino , and J. H. Madans , “The Relation Between Fish Consumption, Death From All Causes, and Incidence of Coronary Heart Disease,” Journal of Clinical Epidemiology 53, no. 3 (2000): 237–244. 10760632 10.1016/s0895-4356(99)00149-3

[9] S. O. Keli , E. J. Feskens , and D. Kromhout , “Fish Consumption and Risk of Stroke,” Stroke 25, no. 2 (1994): 328–332. 8303739 10.1161/01.str.25.2.328

[10] M. C. Morris , J. E. Manson , B. Rosner , J. E. Buring , W. C. Willett , and C. H. Hennekens , “Fish Consumption and Cardiovascular Disease in the Physicians' Health Study: A Prospective Study,” American Journal of Epidemiology 142, no. 2 (1995): 166–175. 7598116 10.1093/oxfordjournals.aje.a117615

[11] A. J. Orencia , M. L. Daviglus , A. R. Dyer , R. B. Shekelle , and J. Stamler , “Fish Consumption and Stroke in Men: 30‐year Findings of the Chicago Western Electric Study,” Stroke 27, no. 2 (1996): 204–209. 8571410 10.1161/01.str.27.2.204

[12] J. Dyerberg , “Eicosapentaenoic Acid and Prevention of Thrombosis and Atherosclerosis?,” Lancet 312, no. 8081 (1978): 117–119. 10.1016/s0140-6736(78)91505-2 78322

[13] T. Terano , A. Hirai , T. Hamazaki , et al., “Effect of Oral Administration of Highly Purified Eicosapentaenoic Acid on Platelet Function, Blood Viscosity and Red Cell Deformability in Healthy Human Subjects,” Atherosclerosis 46, no. 3 (1983): 321–331. 6303363 10.1016/0021-9150(83)90181-8

[14] H. R. Knapp and G. A. FitzGerald , “The Antihypertensive Effects of Fish Oil,” New England Journal of Medicine 320, no. 16 (1989): 1037–1043. 2648152 10.1056/NEJM198904203201603

[15] L. H. Storlien , E. W. Kraegen , D. J. Chisholm , G. L. Ford , D. G. Bruce , and W. S. Pascoe , “Fish Oil Prevents Insulin Resistance Induced by High‐Fat Feeding in Rats,” Science 237, no. 4817 (1987): 885–888. 3303333 10.1126/science.3303333

[16] A. T. Hostmark , T. Bjerkedal , P. Kierulf , H. Flaten , and K. Ulshagen , “Fish Oil and Plasma Fibrinogen,” British Medical Journal 297, no. 6642 (1988): 180–181. 3044509 10.1136/bmj.297.6642.180 PMC1834234

[17] N. Kromann and A. Green , “Epidemiological Studies in the Upernavik District, Greenland: Incidence of Some Chronic Diseases 1950–1974,” Acta Medica Scandinavica 208, no. 1–6 (1980): 401–406. 7457208

[18] S. Suda , T. Katsumata , S. Okubo , et al., “Low Serum n‐3 Polyunsaturated Fatty Acid/n‐6 Polyunsaturated Fatty Acid Ratio Predicts Neurological Deterioration in Japanese Patients With Acute Ischemic Stroke,” Cerebrovascular Diseases 36, no. 5–6 (2013): 388–393. 24248098 10.1159/000355683

[19] J. M. Bamford and C. P. Warlow , “Evolution and Testing of the Lacunar Hypothesis,” Stroke 19, no. 9 (1988): 1074–1082. 3046071 10.1161/01.str.19.9.1074

[20] C. Gandolfo , C. Caponnetto , M. Sette , D. Santoloci , and C. Loeb , “Risk Factors in Lacunar Syndromes: A Case‐Control Study,” Acta Neurologica Scandinavica 77, no. 1 (1988): 22–26. 3354307 10.1111/j.1600-0404.1988.tb06968.x

[21] T.‐J. Song , Y. Chang , M. J. Shin , J. H. Heo , and Y. J. Kim , “Low Levels of Plasma Omega 3‐polyunsaturated Fatty Acids Are Associated With Cerebral Small Vessel Diseases in Acute Ischemic Stroke Patients,” Nutrition Research 35, no. 5 (2015): 368–374. 25921638 10.1016/j.nutres.2015.04.008

[22] R. Chowdhury , S. Stevens , D. Gorman , et al., “Association Between Fish Consumption, Long Chain Omega 3 Fatty Acids, and Risk of Cerebrovascular Disease: Systematic Review and Meta‐Analysis,” British Medical Journal 345 (October 2012): e6698, 10.1136/bmj.e6698. 23112118 PMC3484317

[23] A. S. Abdelhamid , T. J. Brown , J. S. Brainard , et al., “Omega 3 Fatty Acids for the Primary and Secondary Prevention of Cardiovascular Disease,” Cochrane Database of Systematic Reviews 29, no. 3 (2020): CD003177, 10.1002/14651858.CD003177.pub3. PMC7049091 32114706

[24] Y.‐J. Kim , O. Y. Kim , Y. Cho , et al., “Plasma Phospholipid Fatty Acid Composition in Ischemic Stroke: Importance of Docosahexaenoic Acid in the Risk for Intracranial Atherosclerotic Stenosis,” Atherosclerosis 225, no. 2 (2012): 418–424. 23044095 10.1016/j.atherosclerosis.2012.09.007

[25] Y. Ueno , N. Miyamoto , K. Yamashiro , R. Tanaka , and N. Hattori , “Omega‐3 Polyunsaturated Fatty Acids and Stroke Burden,” International Journal of Molecular Sciences 20, no. 22 (November 2019): 5549, 10.3390/ijms20225549. 31703271 PMC6888676

[26] S. Bhat , S. Sarkar , D. Zaffar , P. Dandona , and R. R. Kalyani , “Omega‐3 Fatty Acids in Cardiovascular Disease and Diabetes: A Review of Recent Evidence,” Current Cardiology Reports 25 (2023): 51–65, 10.1007/s11886-022-01831-0. 36729217

[27] A. Cupino , G. Fraser , S. Knutsen , et al., “Are Total Omega‐3 and Omega‐6 Polyunsaturated Fatty Acids Predictors of Fatal Stroke in the Adventist Health Study 2 Prospective Cohort?,” PLoS One 17, no. 9 (2022): e0274109, 10.1371/journal.pone.0274109. 36084005 PMC9462555

[28] H. Iso , K. M. Rexrode , M. J. Stampfer , et al., “Intake of Fish and Omega‐3 Fatty Acids and Risk of Stroke in Women,” Journal of the American Medical Association 285, no. 3 (2001): 304–312. 11176840 10.1001/jama.285.3.304

[29] Y. Park , S. Park , H. Yi , et al., “Low Level of n‐3 Polyunsaturated Fatty Acids In Erythrocytes is a Risk Factor for Both Acute Ischemic and Hemorrhagic Stroke in Koreans,” Nutrition Research 29, no. 12 (2009): 825–830. 19963154 10.1016/j.nutres.2009.10.018

[30] W.‐S. Ryu , S.‐W. Jeong , and D.‐E. Kim , “Total Small Vessel Disease Burden and Functional Outcome in Patients With Ischemic Stroke,” PLoS One 15, no. 11 (2020): e0242319. 33180837 10.1371/journal.pone.0242319 PMC7660472

[31] J. J. Ge , Y. Q. Xing , H. X. Chen , L. J. Wang , and L. Cui , “Analysis of Young Ischemic Stroke Patients in Northeast China,” Annals of Translational Medicine 8, no. 1 (2020): 3. 32055594 10.21037/atm.2019.12.72 PMC6995736

[32] B. Rawlley , S. Marchina , S. P. Cappucci , et al., “Investigation on Gender Differences in Leadership of Stroke‐Related Clinical Trials,” Stroke 54 (2023): 295–303. 36300372 10.1161/STROKEAHA.122.039173

